# Nomogram Prediction Model and Prognostic Comparison of Cervical Clear Cell Carcinoma and Cervical Endometrioid Adenocarcinoma: A SEER Database Study

**DOI:** 10.1002/cam4.71699

**Published:** 2026-03-11

**Authors:** Jimiao Huang, Xiaoyan Li, Yiling Zhuang, Zhonghai Zhang, Junjie You, Hongwei Zhang, Jiamin Chen, Nianquan You, Rui Tang, Wuyuan Pan, Ruqi Fang, Suyu Li, Xiangqin Zheng

**Affiliations:** ^1^ College of Clinical Medicine for Obstetrics & Gynecology and Pediatrics Fujian Medical University, Fujian Maternity and Child Health Hospital Fuzhou China; ^2^ Department of Medicine and Nursing Sanming Medical and Technological College, Clinical Medicine Sanming China; ^3^ Department of Anesthesiology Fujian Medical University Fuzhou China; ^4^ Department of Molecular Biology and Biochemistry Rutgers University‐New Brunswick School of Arts and Sciences New Brunswick New Jersey USA

**Keywords:** cervical clear cell adenocarcinoma, cervical Endometrioid adenocarcinoma, prognostic nomogram, SEER database

## Abstract

**Background:**

Cervical clear cell adenocarcinoma (CCAC) and cervical endometrioid adenocarcinoma (CEAC) are rare and aggressive non‐HPV‐associated malignancies. Despite their histological similarities, these subtypes demonstrate distinct biological behaviors, presenting challenges in treatment and prognosis.

**Objective:**

To develop and validate a multivariable prognostic model for CCAC and CEAC, utilizing the SEER database to enhance clinical decision‐making.

**Methods:**

A total of 775 CEAC and 421 CCAC cases were analyzed using a multivariable nomogram. Patients were randomly allocated to model‐development (*n* = 838) and validation (*n* = 358) cohorts in a 7:3 ratio. The model's performance was evaluated through AUC, Brier score, and Calibration. Decision Curve Analysis (DCA) and Clinical Impact Curve (CIC) were assessed in both development and internal validation cohorts.

**Results:**

The model exhibited excellent calibration and discrimination in predicting overall survival (OS). In the development cohort, the 12‐ and 24‐month prediction models had AUCs of 0.894 (95% CI: 0.860–0.928) and 0.857 (95% CI: 0.821–0.892), respectively. In the internal validation cohort, the 12‐ and 24‐month models achieved AUCs of 0.814 (95% CI: 0.788–0.840) and 0.798 (95% CI: 0.775–0.822), respectively. The model effectively stratified patients into low‐, intermediate‐, and high‐risk groups, with significantly different median survival times (*p* < 0.0001). DCA and CIC further validated the model's clinical utility.

**Conclusion:**

We developed a robust nomogram for quantifying OS risk in CCAC and CEAC patients. This model provides clinicians with a tool for identifying high‐risk patients and implementing timely interventions.

## 1 Introduction

Cervical clear cell adenocarcinoma (CCAC) and cervical endometrioid adenocarcinoma (CEAC) are two distinct histological subtypes of cervical cancer, with unique clinical and pathological features. CCAC is a relatively rare subtype, and its pathogenesis remains partially understood. The IECC classifies both CCAC and CEAC as non‐HPV‐associated cervical cancers, and this was reinforced in the 2020 WHO classification [1]. The typical pathological features of these two types are distinct: CCAC is characterized by solid, papillary, and/or tubular structures with polygonal cells that exhibit high nuclear atypia but uniformity [1]. CEAC, on the other hand, presents with endometrioid morphology featuring “confirmatory endometrioid features” (at least focal, well‐defined low‐grade endometrioid glandular lining by columnar cells, with pseudostratified nuclei showing no more than moderate atypia, with or without squamous differentiation and/or endometriosis) [2]. Both types are extremely rare, with CCAC accounting for 3% and CEAC for just 1.1% of the 371 reported cases of cervical adenocarcinoma [1]. Another study reported on 47 cases where CCAC developed following utero exposure to diethylstilbestrol (DES), with the age distribution of CCAC patients showing two distinct peaks: One in younger women (average age 26 years) and another in older women (average age 71 years). Among women exposed to DES, the incidence of CCAC from birth to age 39 is approximately 1.6 per 1000 [3]. In patients not exposed to DES, CACC accounts for nearly 3%–10% of cervical adenocarcinomas [4, 5, 6], suggesting that genetic or exogenous risk factors also contribute to carcinogenesis [5]. Similarly, primary CEAC is believed to be associated with endometriosis, although its precursor lesions are not well defined [7]. Their non‐HPV association presents significant challenges for early HPV screening. Nevertheless, high‐quality cytopathology remains valuable, enabling early detection in most cases.

A multivariate analysis of non‐HPV‐associated adenocarcinomas (NHPVAs) revealed that overall survival (OS) is significantly influenced by advanced stage, age, high grade, and tumor size [5, 8]. While another study compared 15 cases of FIGO stage IB‐IIB CCAC with 444 cases of squamous cell carcinoma (SCC) and 59 cases of non‐clear cell adenocarcinoma. It found that 80% of CCAC cases exhibited an endophytic growth pattern and were more likely to infiltrate deeply into the cervix and extend to the uterine corpus (*p* < 0.001). CCAC also showed higher rates of parametrial involvement (40%) and pelvic lymph node metastasis (47%), with a lower 5‐year survival rate (67%) compared to SCC (80%) and non‐clear cell adenocarcinoma (77%) [9]. Additionally, positive margins and lympho‐vascular invasion (*p* < 0.05) were also associated with poorer prognosis. Moreover, TP53 mutations are significantly more common in HPV‐independent (HPVI) tumors compared to HPV‐associated (HPVA) tumors (*p* < 0.001) [10], underscoring a genetic distinction between these two adenocarcinoma types. Given the distinct clinicopathological features and biological behaviors of CCAC and CEAC, it is crucial to evaluate and compare their prognostic risk factors to improve patient outcomes and develop individualized treatment strategies.

Several SEER‐based studies have examined prognostic factors for cervical adenocarcinomas, including a recent CCAC‐specific analysis [11], yet these investigations have been limited to single histological subtypes without direct comparative analysis. Our study addresses this gap by developing an integrated nomogram that simultaneously predicts OS for both CCAC and CEAC patients. Furthermore, we comprehensively evaluate its clinical utility through Decision Curve Analysis and Clinical Impact Curve assessment, providing a robust risk stratification tool for these rare non‐HPV‐associated malignancies.

## 2 Materials and Methods

### 2.1 Study Participants

The data for this study cohort were sourced from the SEER 17 Regs incidence study data published in November 2021, covering data changes from 2000 to 2019. These data were extracted from the SEER database, which is a plentiful and worthy resource for cancer‐related information, including information from 17 registration offices in the United States, including nearly 30% of the United States population [12]. Due to its detailed records and extensive coverage, the SEER database has become an important tool in cancer research, providing a rich data foundation for our studies.

The SEER database has always been publicly accessible, and the Institutional Review Board (IRB) has determined that this study did not require approval. Nevertheless, to ensure adherence to ethical principles, we obtained the necessary authorization and permissions for accessing and using the data from the SEER database.

### 2.2 Data Collection

**FIGURE 1:**
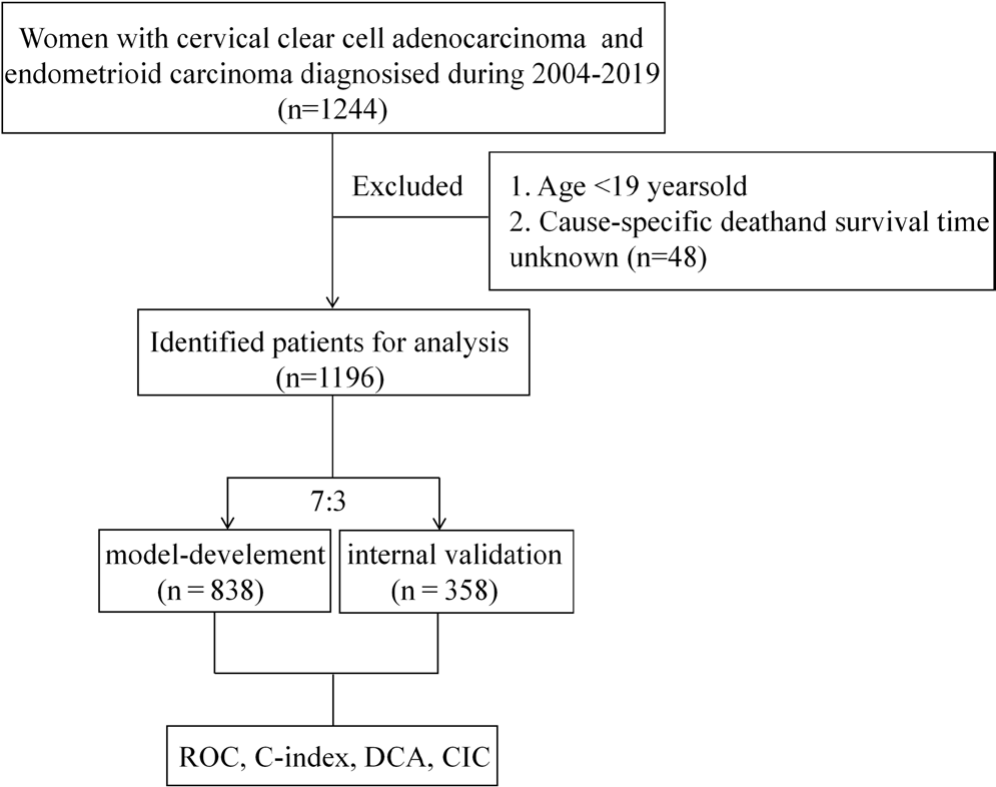
Study design and flowchart. Abbreviations: ROC, Receiver Operating Characteristic; DCA, Decision Curve Analysis; CIC, Clinical Impact Curve; NRI, Net Reclassification Improvement; IDI, Integrated Discrimination Improvement.

We selected patients with primary tumors located in the cervix from the SEER database (version 8.4.3) during the period 2004 to 2019. THE R CODE USED FOR DATA EXTRACTION AND CLEANING IS AVAILABLE FROM THE CORRESPONDING AUTHOR UPON REASONABLE REQUEST. To ensure the availability of the research results, we implemented stringent inclusion and exclusion rules. Patients included in this research must satisfy the following criteria: Diagnosis of a primary tumor located in the cervix, with ICD‐O‐3 histological type codes of 8310 for CCAC or 8380 for CEAC. Additionally, data on patient survival months and vital status were required. To ensure the integrity and accuracy of the research and to enhance the credibility of the results, exclusion criteria included patients under 19 years of age, missing survival time data, and missing cause‐specific death and survival time (CSS) and vital status information. The patient selection process is detailed in Figure 1.

The objective of this study was to develop a nomogram model to predict OS for patients with CCAC and CEAC. The variables included in the study were race (white/others), histological type (CCAC/CEAC), age (19–45, 46–60, > 60 years), stage (I, II, III, IV, unknown), grade (I, II, III, IV), tumor size (< 2 cm, 2–4 cm, > 4 cm, unknown), distant metastasis (yes/no), surgery (yes, no/unknown), radiation (yes, no/unknown), chemotherapy (yes, no/unknown), and regional lymph nodes (positive, Unknown/Negative). BASELINE STAGING DATA FOR THE INCLUDED PATIENTS WERE CLASSIFIED ACCORDING TO THE FOLLOWING SYSTEMS: SEER‐MODIFIED AJCC (3RD EDITION), DERIVED SEER COMBINED STAGE GROUP, DERIVED AJCC STAGE GROUP (6TH AND 7TH EDITIONS), AND DERIVED EOD 2018 STAGE GROUP. These factors will contribute to a comprehensive assessment and prediction of patient survival prognosis.

### 2.3 Statistical Analysis

The dataset of 1196 patients with cervical CCAC and CEAC was divided into a model‐development (*n* = 838) and validation (*n* = 358) cohorts in a 7:3 ratio using the “create Data Partition” function from the “caret” package in R. To compare categorical variables, we employed the chi‐square test and Pearson's chi‐square test for the analysis between different groups. For variables adhering to a Gaussian distribution, means ± standard deviations were analyzed. For variables with a skewed distribution, data were represented using interquartile ranges (Q1–Q3). The nomogram prediction model was established following three steps. Firstly, univariate and multivariate regression analyses were performed to determine features strongly correlated with OS. Secondly, to enhance predictive accuracy and interpretability, and to ensure optimal model fitting, we employed three robust statistical methods—R‐square (R2), Mallows’ Cp, and Least Absolute Shrinkage and Selection Operator (LASSO)—to select variables from the main cohort. Cross‐validation was employed to ascertain the appropriate tuning parameter (λ) in the LASSO logistic regression, allowing LASSO to identify the most significant features. Thirdly, the cohort was divided into a model‐development and an internal validation cohort, and variables were included in the training model based on the results from R‐square, Mallows’ Cp, and LASSO. Subsequently, the model was assessed through receiver operating characteristic (ROC) curves, area under the curve (AUC), values of Harrell's concordance index (C‐index) at different time points, combined with Decision Curve Analysis (DCA) and Clinical Impact Curves (CIC). The model was then internally validated. Data evaluation in this study was performed with the R statistical software (The R Foundation, https://www.R‐project.org). Statistical significance was assessed using two‐tailed tests.

## 3 Results

### 3.1 Clinical Characteristics

From 2004 to 2019, a total of 1196 patients with cervical CCAC and CEAC were included in this study. According to the ICD‐O‐3 histological classification, these patients were divided into the CCAC group and the CEAC group. The characteristics of the group were shown in Table 1. The analysis showed that in younger women (< 60 years), the incidence of CEAC was significantly higher than that of CCAC, while the incidence was opposite in older women (> 60 years) (*p* < 0.001). The incidence of CEAC was highest in the middle‐aged group (40.5%), whereas CCAC was highest in the older group (46.6%). Overall, patients with CEAC had a higher survival rate compared to those with CCAC (74.6% vs. 54.2%).

**TABLE 1:** Characteristics of variables included in the group.

Characteristics	Total (*n* = 1,196)	Endometrioid adenocarcinoma (*n* = 775)	Clear cell carcinomama (*n* = 421)	*P*‐value
Race, *n* (%)				0.007
White	973 (81.4)	648 (83.6)	325 (77.2)	
Others	223 (18.6)	127 (16.4)	96 (22.8)	
Age, *n* (%)				< 0.001
19–45	380 (31.8)	279 (36)	101 (24)	
46–60	438 (36.6)	314 (40.5)	124 (29.5)	
> 60	378 (31.6)	182 (23.5)	196 (46.6)	
Stage, *n* (%)				< 0.001
I	530 (44.3)	407 (52.5)	123 (29.2)	
II	113 (9.4)	72 (9.3)	41 (9.7)	
III	177 (14.8)	98 (12.6)	79 (18.8)	
IV	106 (8.9)	59 (7.6)	47 (11.2)	
Unknown	270 (22.6)	139 (17.9)	131 (31.1)	
Grade, *n* (%)				< 0.001
I	260 (21.7)	250 (32.3)	10 (2.4)	
II	336 (28.1)	303 (39.1)	33 (7.8)	
III	231 (19.3)	91 (11.7)	140 (33.3)	
IV	369 (30.9)	131 (16.9)	238 (56.5)	
Tumor size, *n* (%)				< 0.001
< 2 cm	270 (22.6)	176 (22.7)	94 (22.3)	
2–4 cm	342 (28.6)	244 (31.5)	98 (23.3)	
> 4 cm	333 (27.8)	185 (23.9)	148 (35.2)	
Unknown	251 (21.0)	170 (21.9)	81 (19.2)	
Regional lymph nodes, *n* (%)				0.249
Positive	147 (12.3)	89 (11.5)	58 (13.8)	
Unknown/Negative	1,049 (87.7)	686 (88.5)	363 (86.2)	
Surgery, *n* (%)				< 0.001
Yes	861 (72.0)	601 (77.5)	260 (61.8)	
No/Unknown	335 (28.0)	174 (22.5)	161 (38.2)	
Radiation, *n* (%)				< 0.001
Yes	632 (52.8)	370 (47.7)	262 (62.2)	
No/Unknown	564 (47.2)	405 (52.3)	159 (37.8)	
Chemotherapy, *n* (%)				< 0.001
Yes	516 (43.1)	294 (37.9)	222 (52.7)	
No/Unknown	680 (56.9)	481 (62.1)	199 (47.3)	
Bone metastasis, *n* (%)				0.091
Yes	10 (0.8)	5 (0.6)	5 (1.2)	
No	690 (57.7)	432 (55.7)	258 (61.3)	
Unknown	496 (41.5)	338 (43.6)	158 (37.5)	
Brain metastasis, *n* (%)				0.004
Yes	4 (0.3)	0 (0)	4 (1)	
No	696 (58.2)	437 (56.4)	259 (61.5)	
Unknown	496 (41.5)	338 (43.6)	158 (37.5)	
Liver metastasis, *n* (%)				0.07
Yes	12 (1.0)	6 (0.8)	6 (1.4)	
No	687 (57.4)	430 (55.5)	257 (61)	
Unknown	497 (41.6)	339 (43.7)	158 (37.5)	
Pulmonary metastasis, *n* (%)				0.013
Yes	30 (2.5)	13 (1.7)	17 (4)	
No	668 (55.9)	424 (54.7)	244 (58)	
Unknown	498 (41.6)	338 (43.6)	160 (38)	
Distance metastasis, *n* (%)				0.004
Yes	45 (3.8)	20 (2.6)	25 (5.9)	
No	1,151 (96.2)	755 (97.4)	396 (94.1)	
Css, *n* (%)				< 0.001
Alive or dead of other cause	911 (76.2)	636 (82.1)	275 (65.3)	
Dead (attributable to this cancer)	285 (23.8)	139 (17.9)	146 (34.7)	
Survival months, Median (IQR)	60.0 (21.0, 115.0)	75.0 (32.5, 129.0)	33.0 (12.0, 86.0)	< 0.001
Vital status, *n* (%)				< 0.001
Alive	806 (67.4)	578 (74.6)	228 (54.2)	
Dead	390 (32.6)	197 (25.4)	193 (45.8)	

In the analysis of distant organ metastasis, the highest rate was observed in the lungs, followed by the liver, bones, and brain. Overall, the organ metastasis rate for CCAC (5.9%) was significantly higher than that for CEAC (2.6%). Notably, the lung metastasis rate for CCAC and CEAC was 4% vs. 1.4%. Additionally, significant differences were found between CCAC and CEAC patients in terms of race, grade, stage, tumor size, CSS, treatment methods, and OS. The grades and stages of CCAC were higher than those of CEAC. Nevertheless, there was no meaningful statistical distinction between the cohorts in the number of regional lymph nodes.

In this study, 838 patients were assigned to the model development cohort, while 358 patients were assigned to the internal validation cohort. The detailed characteristics of the two cohorts are shown in Table 2. The median survival months for the model development and internal validation cohorts were 60.0 (21.0, 115.0) and 60.5 (22.2, 113.0), with no statistically significant difference between them (*p* = 0.706). Moreover, there were no notable statistical distinctions between the cohorts regarding distant metastasis, age, stage, and tumor size (*p* > 0.05).

**TABLE 2:** Characteristics of variables included in the model‐development and internal validation cohorts.

Characteristics	Total (*n* = 1,196)	The model‐development Cohort (*n* = 838)	The internal validation Cohort (*n* = 358)	*P*‐value
Histological type				0.79
Endometrioid carcinoma	775 (64.8)	541 (64.6)	234 (65.4)	
Clear cell adenocarcinma	421 (35.2)	297 (35.4)	124 (34.6)	
Race, *n* (%)				0.543
White	973 (81.4)	678 (80.9)	295 (82.4)	
Others	223 (18.6)	160 (19.1)	63 (17.6)	
Age, *n* (%)				0.488
19–45	380 (31.8)	261 (31.1)	119 (33.2)	
46–60	438 (36.6)	316 (37.7)	122 (34.1)	
> 60	378 (31.6)	261 (31.1)	117 (32.7)	
Stage, *n* (%)				0.675
I	530 (44.3)	366 (43.7)	164 (45.8)	
II	113 (9.4)	86 (10.3)	27 (7.5)	
III	177 (14.8)	124 (14.8)	53 (14.8)	
IV	106 (8.9)	75 (8.9)	31 (8.7)	
Unknown	270 (22.6)	187 (22.3)	83 (23.2)	
Grade, *n* (%)				0.712
I	260 (21.7)	176 (21)	84 (23.5)	
II	336 (28.1)	233 (27.8)	103 (28.8)	
III	231 (19.3)	165 (19.7)	66 (18.4)	
IV	369 (30.9)	264 (31.5)	105 (29.3)	
Tumor size, *n* (%)				0.367
< 2 cm	270 (22.6)	189 (22.6)	81 (22.6)	
2–4 cm	342 (28.6)	228 (27.2)	114 (31.8)	
> 4 cm	333 (27.8)	238 (28.4)	95 (26.5)	
Unknown	251 (21.0)	183 (21.8)	68 (19)	
Regional lymph nodes, *n* (%)				0.248
Positive	147 (12.3)	109 (13)	38 (10.6)	
Unknown/Negative	1,049 (87.7)	729 (87)	320 (89.4)	
Surgery, *n* (%)				0.421
Yes	861 (72.0)	609 (72.7)	252 (70.4)	
No/Unknown	335 (28.0)	229 (27.3)	106 (29.6)	
Radiation, *n* (%)				0.513
Yes	632 (52.8)	448 (53.5)	184 (51.4)	
No/Unknown	564 (47.2)	390 (46.5)	174 (48.6)	
Chemotherapy, *n* (%)				0.086
Yes	516 (43.1)	375 (44.7)	141 (39.4)	
No/Unknown	680 (56.9)	463 (55.3)	217 (60.6)	
Distance metastasis, *n* (%)				0.626
Yes	45 (3.8)	33 (3.9)	12 (3.4)	
No	1,151 (96.2)	805 (96.1)	346 (96.6)	
Css, *n* (%)				0.732
Alive or dead of other cause	911 (76.2)	636 (75.9)	275 (76.8)	
Dead (attributable to this cancer)	285 (23.8)	202 (24.1)	83 (23.2)	
Survival months, Median (IQR)	60.0 (21.0, 115.0)	60.0 (21.0, 115.0)	60.5 (22.2, 113.0)	0.706
Vital status, *n* (%)				0.972
Alive	806 (67.4)	565 (67.4)	241 (67.3)	
Dead	390 (32.6)	273 (32.6)	117 (32.7)	

### 3.2 Univariate and Multivariate Cox Regression Analysis of OS in CCAC and CEAC

Univariate logistic regression analysis demonstrated that histological type, age, stage, grade, tumor size, metastasis, regional lymph nodes, primary site surgery, and chemotherapy were strongly correlated with OS. Further multivariate logistic regression analysis confirmed the independent impact of age, stage, metastasis, primary site surgery, and chemotherapy on OS. Detailed findings from the univariate and multivariate logistic regression analyses are outlined in Table S1.

### 3.3 Variable Selection

**FIGURE 2:**
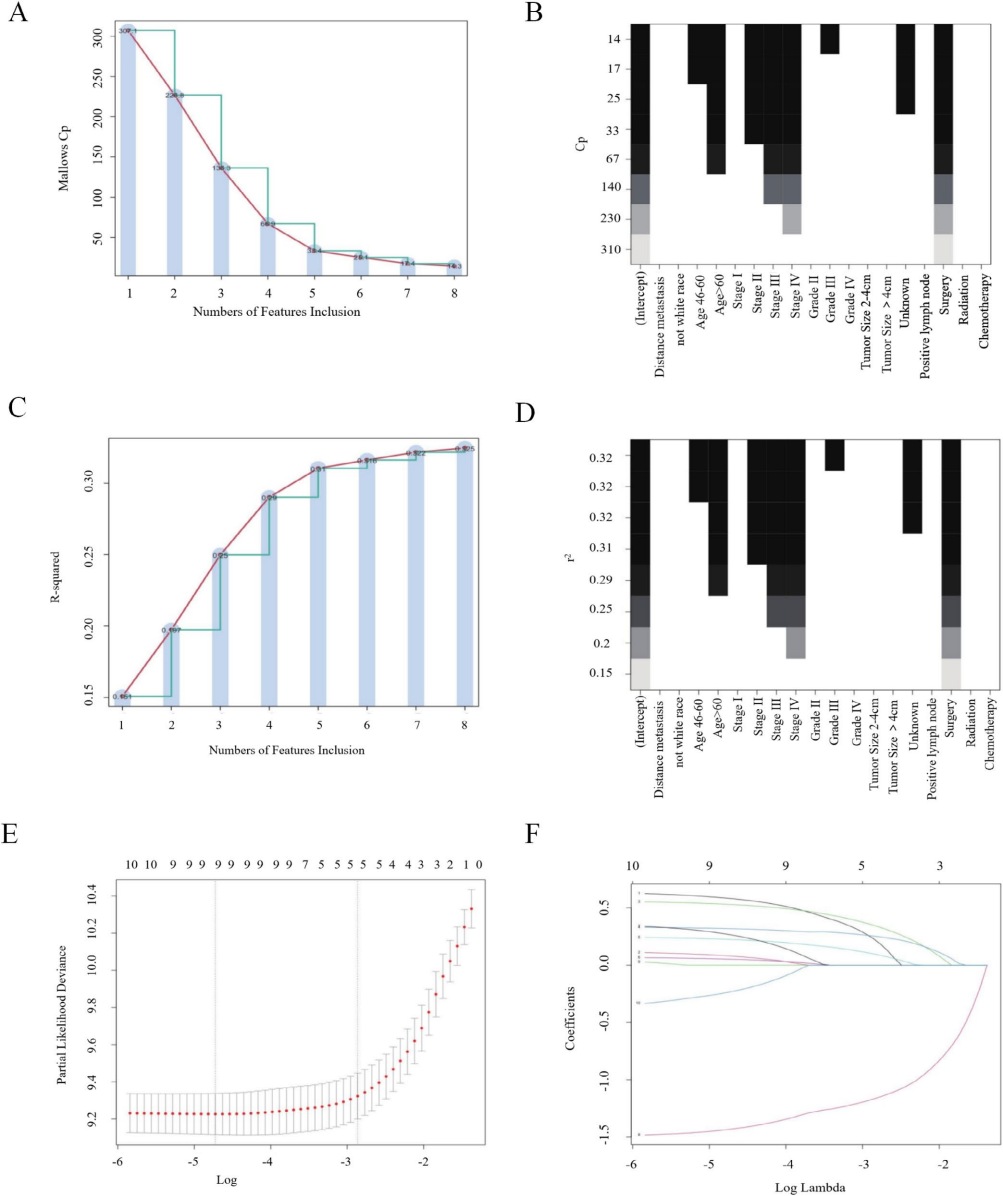
Methods of variable selection. (A, B) Variable selection was performed using Mallows' CP method. (A) The minimum Cp value of 14.3 was observed at the inflection point of the curve. (B) The y‐axis represents the adjusted Cp values, and the x‐axis denotes the variables. (C, D) Variable selection was performed using the R‐squared (R^2^) method. (C) The minimum R^2^ value was 0.325, observed at the inflection point of the segmented line. (D) The y‐axis represents the R^2^ values, and the x‐axis denotes the variables. (E) LASSO regression was used for variable selection, and cross‐validation was employed to determine the tuning parameter (λ). (F) LASSO coefficient profiles for variables related to OS in the model development cohort. Abbreviations: LASSO, Least Absolute Shrinkage and Selection Operator.

To optimize the selection of variables for the model, this study employed three common statistical methods: Mallows' CP, R‐squared (R^2^), and LASSO. Mallows' Cp method also selected five variables: Age, grade, surgery, stage, and tumor size, with a Cp value of 14.3, further indicating the significance of these variables Figure 2A,B. The R‐squared (R^2^) method selected five variables: Age, grade, surgery, stage, and tumor size, with an adjusted maximum R^2^ value of 0.325, reflecting the contribution of these variables to the model's explanatory power Figure 2C,D. The LASSO method selected four variables that were statistically significant: Age, stage, surgery, and metastasis, with a lambda value of 0.057. The coefficient path plots for LASSO are illustrated in Figure 2E,F, illustrating the changes in coefficients for different lambda values. All selected variables exhibited significant statistical differences (*p* < 0.05), validating their importance for the model's predictive and explanatory capabilities.

### 3.4 Development of the Model

**FIGURE 3:**
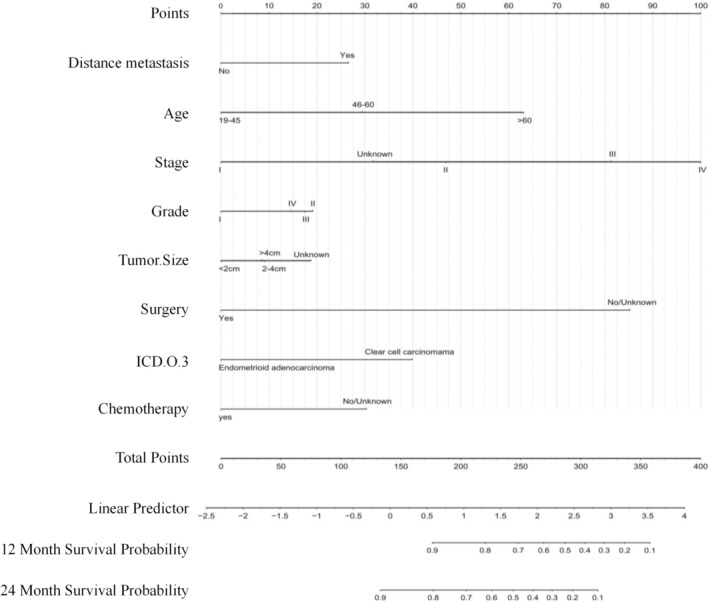
Nomogram for predicting overall survival (OS) in patients with CCAC and CEAC using variables from a model development cohort.

Drawing from the findings of univariate and multivariate regression analyses, as well as the R‐squared (R^2^), Mallows’ Cp, and LASSO methods, a final set of 8 variables was selected for model construction. These variables include: Age, histological type, tumor size, stage, grade, surgery, distant metastasis, and regional lymph nodes. These variables were incorporated into a nomogram model to evaluate its clinical utility Figure 3.

### 3.5 Model Performance

**FIGURE 4:**
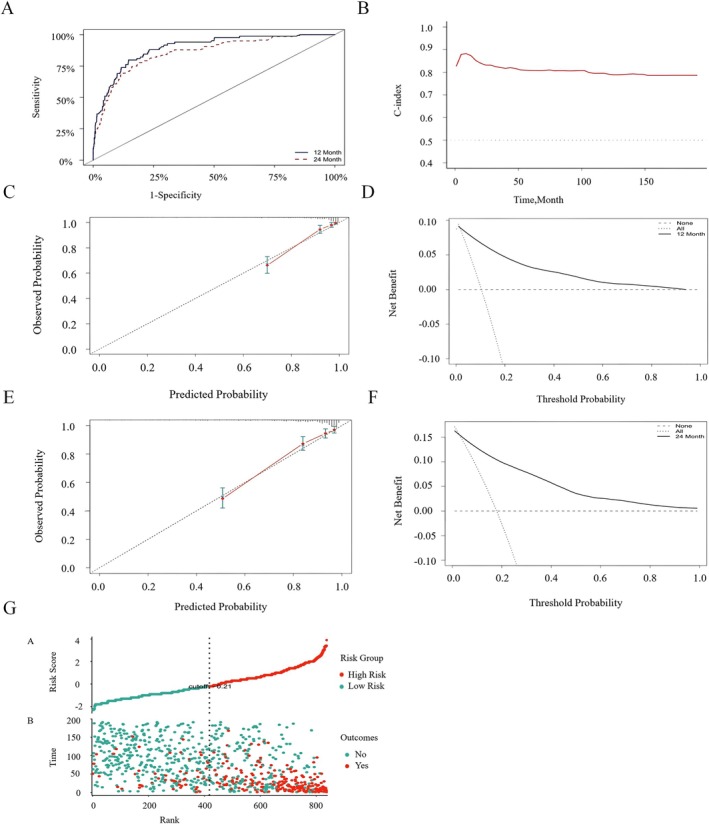
ROC curves, calibration curves, DCA, CIC, and Risk Stratification Plot for OS at 12 and 24 months in the model‐development cohort. (A) The ROC curve shows that the OS discrimination accuracy for the 12‐month prediction model is 0.894 (95% CI: 0.860–0.928), while for the 24‐month prediction model, it is 0.857 (95% CI: 0.821–0.892). (B) Calibration curves for OS at different time points. (C) The close alignment of the DCA curves with the reference lines at 12 months. (D) The CIC plot for 12 months emphasizes the model's substantial net benefit across a wide range of threshold probabilities. (E) The close alignment of the DCA curves with the reference lines at 24 months. (F) The CIC plot for 24 months highlights the model's significant net benefit across various threshold probabilities, demonstrating its robust clinical utility. (G) Risk Stratification Plot: Based on their risk scores, patients were divided into low‐ and high‐risk groups. Abbreviations: ROC, Receiver Operating Characteristic; DCA, Decision Curve Analysis; CIC, Clinical Impact Curve.

The model demonstrated excellent calibration performance. In the model development cohort Figure 4, the 12‐month prediction model had a discrimination accuracy of 0.894 (95% CI: 0.860–0.928), while the 24‐month prediction model had a discrimination accuracy of 0.857 (95% CI: 0.821–0.892). The Hosmer–Lemeshow goodness‐of‐fit test showed R^2^ values of 0.064 for the 12‐month model and 0.087 for the 24‐month model. The 12‐ and 24‐month Decision Curve Analysis (DCA) and Clinical Impact Curve (CIC) results are shown in Figure 4C–F, with the DCA calibration curves closely aligning with the reference line. As the true positive CIC curve converges at different risk thresholds, the net benefit significantly increases, highlighting the model's strong clinical utility. Based on their risk scores, patients were divided into low‐risk and high‐risk groups Figure 4G.

**FIGURE 5:**
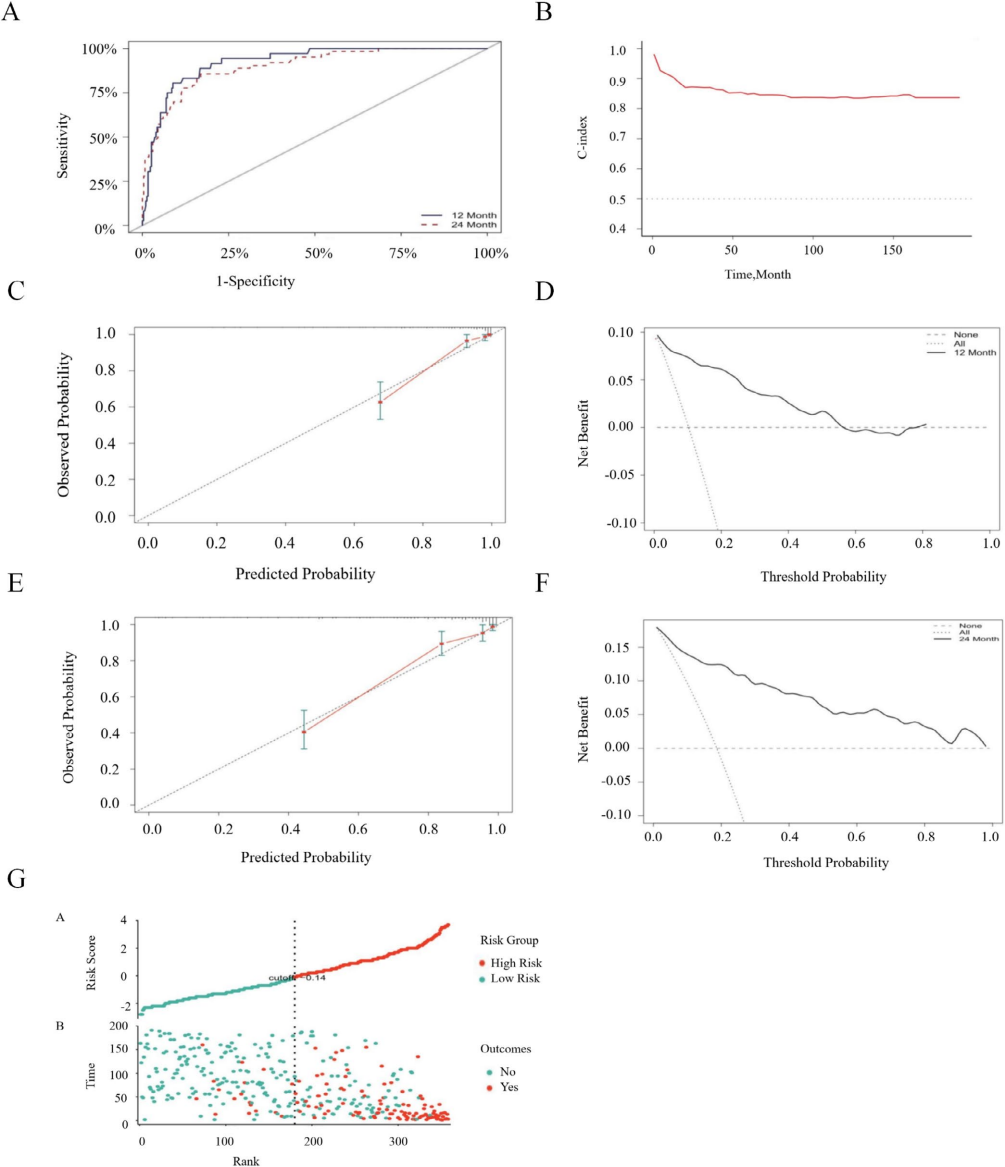
ROC curves, calibration curves, DCA, CIC, and Risk Stratification Plot for OS at 12 and 24 months in the internal validation cohort. (A) In the internal validation cohort, the 12‐month prediction model had an AUC of 0.924 (95% CI: 0.886–0.962) and a Brier score of 0.062, while the 24‐month prediction model had an AUC of 0.901 (95% CI: 0.860–0.942) and a Brier score of 0.087. The lower Brier scores indicate satisfactory model calibration, and the narrow AUC confidence intervals further validate the model's discriminative accuracy. (B) Calibration curves for OS at different time points in the internal validation cohort. (C) The DCA curves closely align with the reference lines at 12 months, indicating strong decision support at this time point. (D) The CIC plot for 12 months demonstrates the model's substantial net benefit across a broad range of threshold probabilities. (E) At 24 months, the DCA curves also align closely with the reference lines, underscoring the model's reliable decision support. (F) The CIC plot for 24 months highlights the model's significant net benefit across various threshold probabilities, showcasing its robust clinical utility. (G) Risk Stratification Plot: Patients were classified into low‐ and high‐risk groups based on their risk scores.

In the internal validation cohort Figure 5, the 12‐month prediction model had an AUC of 0.924 (95% CI: 0.886–0.962) and a Brier score of 0.062, while the 24‐month prediction model had an AUC of 0.901 (95% CI: 0.860–0.942) and a Brier score of 0.087. The lower Brier scores indicate satisfactory model calibration, and the narrow AUC confidence intervals further validate the model's discriminative accuracy. The DCA and CIC results in Figure 5C–F further support the practical utility of this model in clinical settings. Based on the patients' risk scores, they can be divided into low‐risk and high‐risk groups in the training set as well Figure 5G.

### 3.6 Kaplan–Meier Curve

**FIGURE 6:**
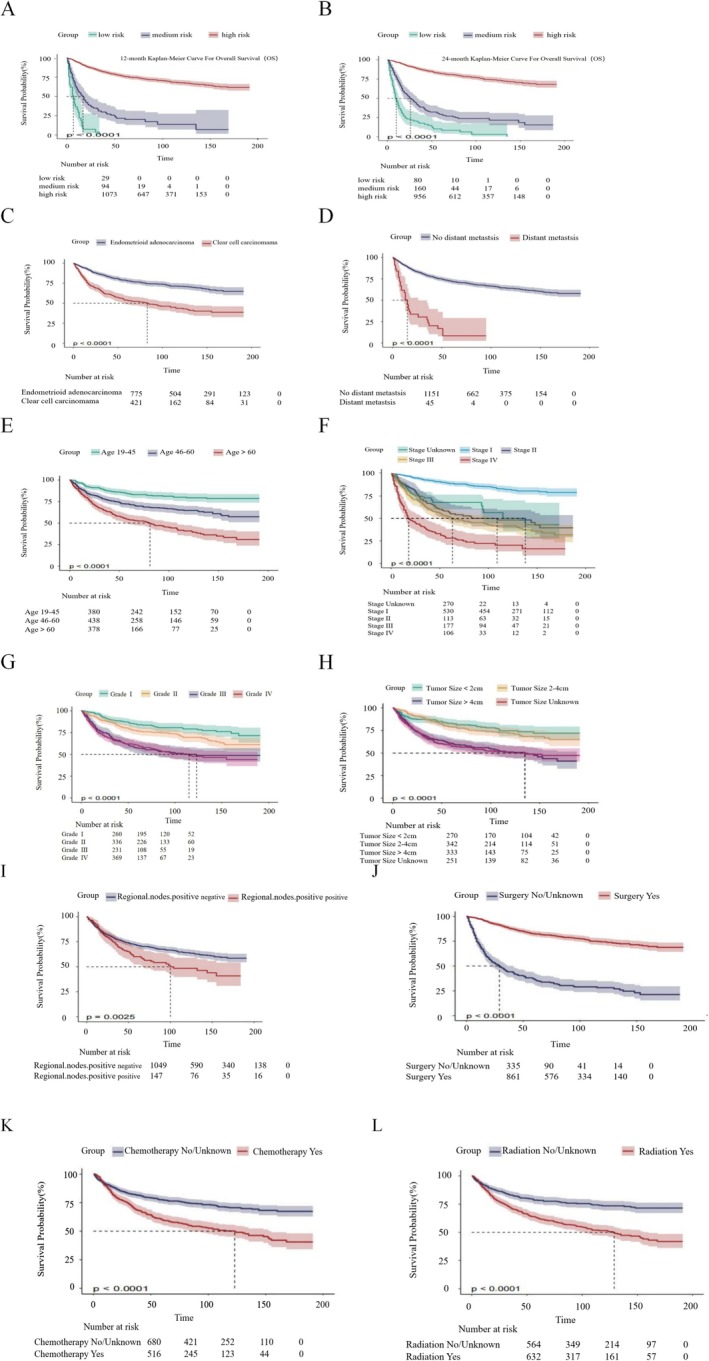
Survival Analysis and Prognostic Factors in CCAC and CEAC. (A,B) Kaplan–Meier survival curves for the 12‐ and 24‐month prediction models showing significantly longer median survival times in the low‐risk group (approximately more than 150 months for the 12‐ and 24‐month models) compared to the medium‐ and high‐risk groups (*p* < 0.0001). (C) The survival curve for CEAC patients is significantly higher than that for CCAC patients, with median survival times of over 200 months vs. 80 months (*p* < 0.0001). (D) Patients without distant organ metastasis have a median survival time of 130 months, whereas those with distant metastasis have a median survival of only 17 months. (E–I) Analysis indicates that younger age, early diagnosis stage, low‐grade cell differentiation, smaller tumor size, and negative regional lymph node are strongly associated with prolonged survival. (J–L) Patients who underwent surgery had a higher overall survival rate, whereas those who received chemotherapy and radiotherapy showed a relative decrease in survival rate. This underscores the effectiveness of surgical intervention in improving prognosis for patients at an early stage.

We divided the predictions (0.4, 0.7) from our predictive model into three tertiles to classify patients into low, medium, and high‐risk groups. The study results indicate that, in both the 12‐ and 24‐month predictive models, the median survival time for the low‐risk group exceeds 150 months. This duration is significantly higher than that of the moderate‐risk and high‐risk groups, with differences being statistically significant (*p* < 0.0001, Figure 6A,B). The survival curve for CEAC is notably higher than that for CCAC patients, with median survival times of over 200 months vs. 80 months (*p* < 0.0001, Figure 6C). Additionally, patients without distant organ metastasis have a significantly extended median survival time of 130 months, compared to only 17 months for those with distant metastasis (Figure 6D). Analysis shows that younger age, early diagnosis stage, low‐grade cell differentiation, smaller tumor size, and negative regional lymph nodes are strongly linked to longer survival (Figure 6E–I). Patients who underwent surgery experienced a higher overall survival rate, while those treated with chemotherapy and radiotherapy had relatively lower survival rates (Figure 6J–L).

### 3.7 Subgroup Analysis for CCAC and CEAC

In this subgroup analysis, the prognosis of CCAC was compared to that of CEAC. Overall, CCAC demonstrated a significantly worse prognosis, with a hazard ratio (HR) of 2.52 (95% CI: 2.06–3.08), indicating a markedly higher risk across all subgroups compared to CEAC (Figure S1). Specific subgroup results showed that younger patients (< 45 years) had an HR of 2.05 (95% CI: 1.19–3.54), and older patients (≥ 60 years) had an HR of 1.64 (95% CI: 1.19–2.25). In patients with advanced‐stage (Stage III–IV) disease, the HR was 1.38 (95% CI: 1.02 ~ 1.89). For patients with tumor sizes greater than 4 cm, the HR was 1.48 (95% CI: 1.13–1.94), and for those with positive lymph nodes, the HR was 1.17 (95% CI: 0.69~1.98). Additionally, for patients who received surgery, the HR for CCAC was 1.51 (95% CI: 1.1~2.07), and for those who underwent radiation therapy, the HR was 1.56 (95% CI: 1.2–2.03). Chemotherapy patients had an HR of 1.61 (95% CI: 1.21–2.14), all showing significantly worse outcomes for CCAC. Notably, for patients with Grade I tumors, the difference in prognosis between CCAC and CEAC was not statistically significant (HR = 0.79, 95% CI: 0.17~3.71).

Additionally, CSS analysis demonstrated patterns consistent with the OS findings, as shown in Figures S2–S5.

## 4 Discussion

Our study analyzed 775 CEAC and 421 CCAC cases from the SEER database to develop a robust nomogram prediction model for OS. The model demonstrated strong predictive performance, with AUCs of 0.894 and 0.857 for the 12‐ and 24‐month prediction models in the development cohort, and 0.924 and 0.901 in the internal validation cohort, respectively. Additionally, the log‐rank test and Kaplan–Meier survival curves confirmed the consistency and reliability of the prognostic stratification, revealing that the median survival time for the intermediate‐ and high‐risk groups was significantly shorter (less than 25 and 10 months) compared to the low‐risk group (more than 150 months, *p* < 0.0001). Patients with distant organ metastasis had a notably worse median survival time compared to those without distant metastasis (20 months vs. 130 months). Additionally, poor prognosis was strongly associated with factors such as older age, high grade, advanced stage, larger tumor size, and positive lymph node involvement. However, surgical intervention was linked to an improvement in OS [6, 13, 14]. Although patients who received chemotherapy and radiotherapy exhibited poorer OS, this observation may be influenced by later‐stage diagnosis and the presence of high‐risk factors. Further stratified analysis is necessary to accurately assess the effectiveness of chemo‐radiotherapy in these cases.

When comparing CCAC and CEAC, the analysis revealed significant differences in survival outcomes, with CCAC demonstrating a consistently poorer prognosis. Across all subgroups—irrespective of the patient's age, stage, tumor size, distant metastasis, lymph node status, or treatment strategies—CCAC was associated with worse outcomes than CEAC, which is consistent with previous literature reports [11, 12, 13, 14, 15]. This trend was particularly pronounced in patients with advanced‐stage disease, larger tumors, and lymph node involvement. However, the prognostic gap between these two cancers may narrow in well‐differentiated tumors. These results reinforce the understanding that CCAC is a more aggressive cancer with a generally poorer prognosis. In contrast, CEAC, despite its generally better prognosis, still requires careful management, particularly in cases presenting in high‐risk groups.

Given that both CCAC and CEAC are classified as non‐HPV‐associated adenocarcinomas, with an HPV infection rate of less than 20%. Thomas et al. reported that only 18% of CCAC patients (6/34) had abnormal Pap smears [16]. Additionally, sensitive tumor markers and distinctive imaging features are lacking. Their rarity and aggressiveness underscore the critical importance of early screening and diagnosis. While 66.7% (4/6) of patients also showed abnormal TCT results [4]. Patients with stage II or higher disease may present with elevated CA125 levels (≥ 30 U/mL) [4]. Vang et al. [15] found that immunohistochemical analysis of 17 cases of CCAC showed positivity for CEA, p53, and CA 125. HER‐2/neu and ER were also expressed to varying degrees [17]. Additionally, the expression level of Ki67 is closely associated with lymph node metastasis in cervical cancer [13]. Most CCACs are endophytic, often presenting with deep cervical infiltration, resulting in a barrel‐shaped cervix; 25% had uterine body infiltration [4]. The cervical fibrous connective tissue limits the growth of cysts, typically leading to well‐defined masses on imaging. These tumors are often highly vascularized, resulting in marked enhancement [18]. Therefore, combining features of cytology, imaging, CCAC and CEAC pathology, and immunohistochemistry, and ensuring early diagnosis are key to improving prognosis.

The selection of the best adjuvant treatment approach for CCAC and CEAC remains controversial. Both CCAC and CEAC may originate from the endometrium. Stolnicu et al. [7] proposed that CCAC could arise from cervical endometriosis or tubal endometrioid metaplasia, which may explain their histological and biological similarities to uterine CCC (UCCC). It has been reported that in endometrial tumors, the OS of UCCC is significantly lower than that of endometrioid carcinoma (UEC) (HR = 12.944, 95% CI = 4.231–39.599, *p* < 0.001). Deep myometrial invasion and lymph node metastasis are particularly important prognostic risk factors. Studies have shown that OS is significantly worse for patients with Stage I–II UCCC compared to UEC, which might be partly due to the lack of adjuvant therapy following surgery in early‐stage patients [19]. Referring to the retrospective studies, comprehensive surgical staging combined with systematic lymphadenectomy and vaginal brachytherapy may serve as adequate adjuvant therapy for stages I and II UCCC [20, 21]. The decision to administer adjuvant therapy for CCAC remains controversial. Some studies suggest that CCAC patients may benefit from adjuvant chemotherapy (paclitaxel + platinum) ± radiotherapy [4]. However, other research indicates that chemotherapy or concurrent chemoradiotherapy has little impact on the prognosis of CCAC, especially for early‐stage patients without any pathological risk factors [3, 22]. This discrepancy is mainly due to the small sample sizes and inconsistent staging in the studies included. Overall, adjuvant therapy for CCAC patients should be individualized based on clinical staging and risk factors. The latest NCCN guidelines for cervical clear cell carcinoma (CCAC) recommend a comprehensive approach to treatment, similar to that for other types of cervical cancer. The standard treatment for early‐stage specific types of cervical adenocarcinoma involves radical hysterectomy with pelvic lymphadenectomy. In cases of advanced disease (Stage III–IV) or high‐risk features, adjuvant treatment with concurrent chemoradiotherapy (CRT) is advised, with the use of external beam radiation therapy (EBRT) combined with chemotherapy. Regarding the choice of chemotherapy regimen, Krivak et al. suggested that TC (paclitaxel and carboplatin) is not inferior to TAP (doxorubicin, cisplatin, and paclitaxel) in terms of progression‐free survival (PFS) and OS, while also being associated with less toxicity [23]. Additionally, for patients with positive lymph nodes or other high‐risk factors, the guidelines suggest considering the addition of brachytherapy to improve local control [24]. This underscores the importance of our study in providing prognostic risk stratification to guide clinical decision‐making. Based on our research findings, risk stratification may provide valuable and individualized guidance for treatment strategies.

This study also underscores the necessity for further exploration of new treatment strategies to improve outcomes for CCAC patients. Highlighted the limitations of current treatment protocols for CCAC, suggesting that the incorporation of targeted therapies and immunotherapy might be necessary to improve outcomes. Preliminary metabolic gene expression clustering analysis has identified TP53, ARID1A, and PIK3CA as the most commonly mutated genes across different origins [25]. This similarity suggests that targeting pathways like PI3K‐AKT–mTOR, DNA repair, and MYC could be effective across various sites [26]. Recent advances in the treatment of advanced cervical cancer have shown significant efficacy with various approaches, including immunotherapy alone or in combination with dual immunotherapy, immunotherapy combined with chemoradiotherapy, and the addition or subtraction of anti‐angiogenic agents. Notably, treatments like the combination of Tisotumab vedotin (TV) with carboplatin (Carbo) in the 723MO study have demonstrated promising results [27, 28, 29, 30, 31]. The exploration of biomarkers for immune‐targeted therapy, such as TMB (Tumor Mutational Burden), MSI (Microsatellite Instability), A27 [32], and PD‐L1, provides valuable guidance for developing new treatment strategies. These biomarkers can help tailor treatments to individual patients, potentially leading to better outcomes.

While prognostic nomograms for cervical cancer—including recent CCAC‐specific models—are established in the literature [33, 34], our study distinguishes itself through a comparative CEAC vs. CCAC framework and the incorporation of DCA/CIC to assess clinical utility. This study is subject to several limitations, some intrinsic to SEER database research. First, the retrospective design using the SEER database inherently carries selection and information biases, including missing data on critical confounders such as HPV infection status, detailed treatment protocols (e.g., radiation dose/fractionation, chemotherapy regimens), and comorbidities that may affect outcomes. Second, the internal validation using SEER data may be influenced by regional and racial differences, as well as unquantifiable variations in medical conditions or treatment protocols due to the limitations of the internal validation dataset. Third, the absence of external multicenter validation cohorts means the model's performance in diverse clinical settings remains unproven. Future analyses and prospective studies of multicenter data with larger sample sizes, more variables, including clinical and non‐clinical factors, and patients of different ethnicities are required to validate our conclusions.

## 5 Conclusion

In summary, the prognostic prediction model developed in this study offers clinicians a practical tool to support risk stratification for patients with clear cell adenocarcinoma of the cervix (CCAC) across various anatomical sites of the reproductive tract. As a retrospective investigation, the clinical applicability of this model requires further validation. Patients diagnosed at an early stage—who are potential candidates for comprehensive surgical staging—generally exhibit favorable outcomes. The model may assist in identifying individuals with differential prognostic profiles, which could inform more tailored follow‐up and surveillance strategies.

## Author Contributions

Xiangqin Zheng, Suyu Li, Jimiao Huang, Xiaoyan Li, and Yiling Zhuang were involved in the design of the study. Junjie You, Hongwei Zhang, Jiamin Chen, Nianquan You, and Rui Tang carried out data collection and analysis. Xiaoyan Li, Jimiao Huang, Yiling Zhuang, and Zhonghai Zhang assisted in drafting the manuscript. Xiangqin Zheng, Wuyuan Pan, Ruqi Fang, and Suyu Li revised the manuscript. All the authors reviewed and approved the final manuscript.

## Funding

This work was supported by Fujian Provincial Natural Science Foundation of China (Grant number: 2024J011054), Joint Funds for the Technology Innovation of Science and Fujian Province (Grant number 2024Y9586), and Fujian Medical University Undergraduate Education and Teaching Research Project (grant number Y24023).

## Ethics Statement

Ethical consent was waived due to the SEER database containing anonymous patient information. All data from the SEER database were open access.

## Conflicts of Interest

The authors declare no conflicts of interest.

## Supporting information


**Data S1:** Supplementary Information.

## Data Availability

The data that support the findings of this study are available in Surveillance, Epidemiology, and End Results (SEER) Program at https://seer.cancer.gov/data/seer‐stat/nov2021/. These data were derived from the following resources available in the public domain: ‐ SEER 17 Regs Custom Data (with additional treatment fields), https://seer.cancer.gov/data‐software/.
