# Supplementary material for: Nomogram Prediction Model and Prognostic Comparison of Cervical Clear Cell Carcinoma and Cervical Endometrioid Adenocarcinoma: A SEER Database Study
Source: Cancer Med. 2026 Mar 11;15(3):e71699. doi: 10.1002/cam4.71699 (PMC12976804; doi:10.1002/cam4.71699)
Supplement: Supplementary file 1 — Data S1: Supplementary Information. [file CAM4-15-e71699-s001.docx]

Supplementary Material

# Supplementary Figures and Tables

## Supplementary Figures


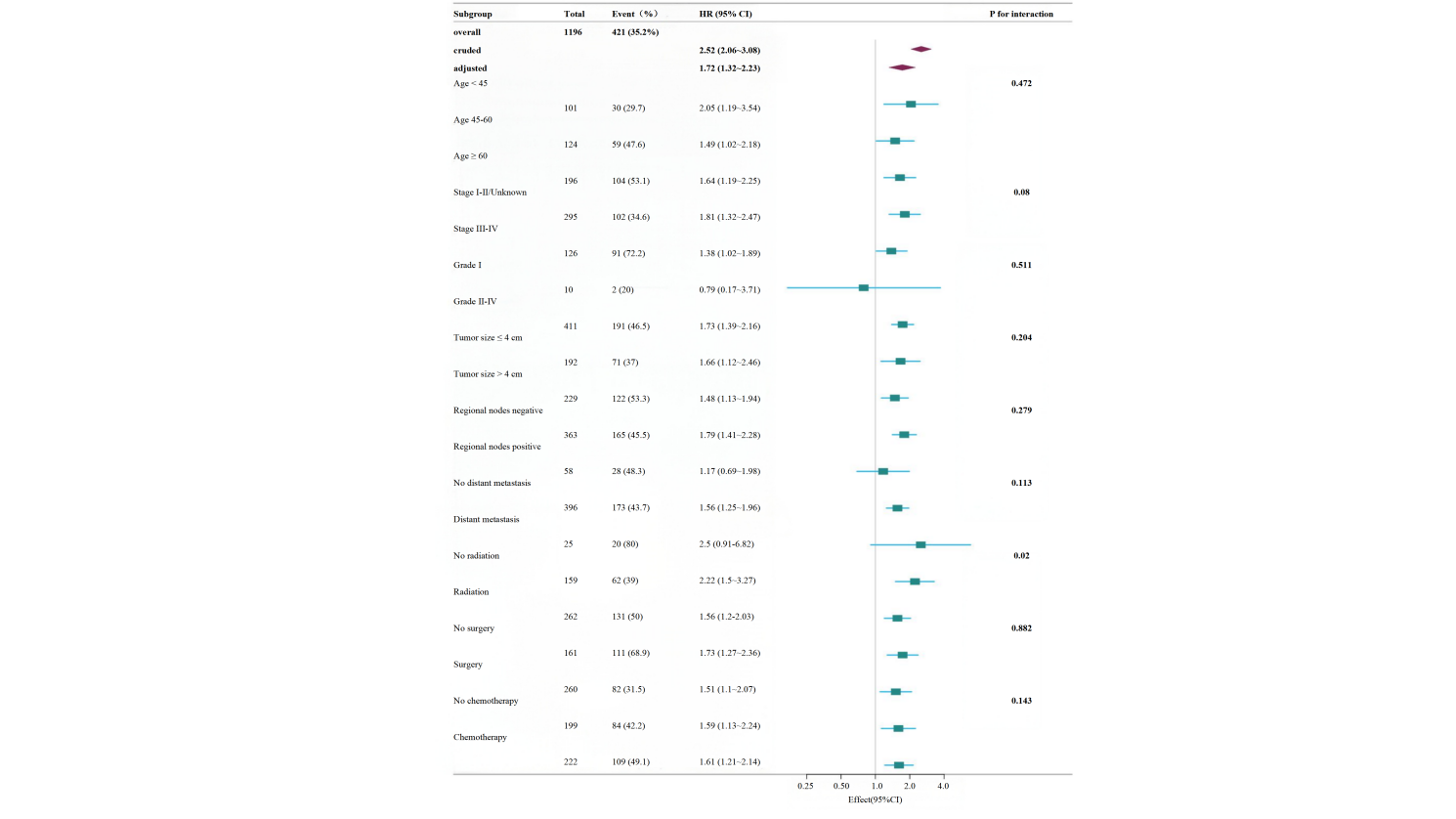


**Supplementary Figure 1.** Subgroup analysis shows that CCAC has a significantly worse prognosis, with a hazard ratio (HR) of 2.52 (95% CI: 2.06–3.08), indicating a markedly higher risk across all subgroups compared to CEAC.


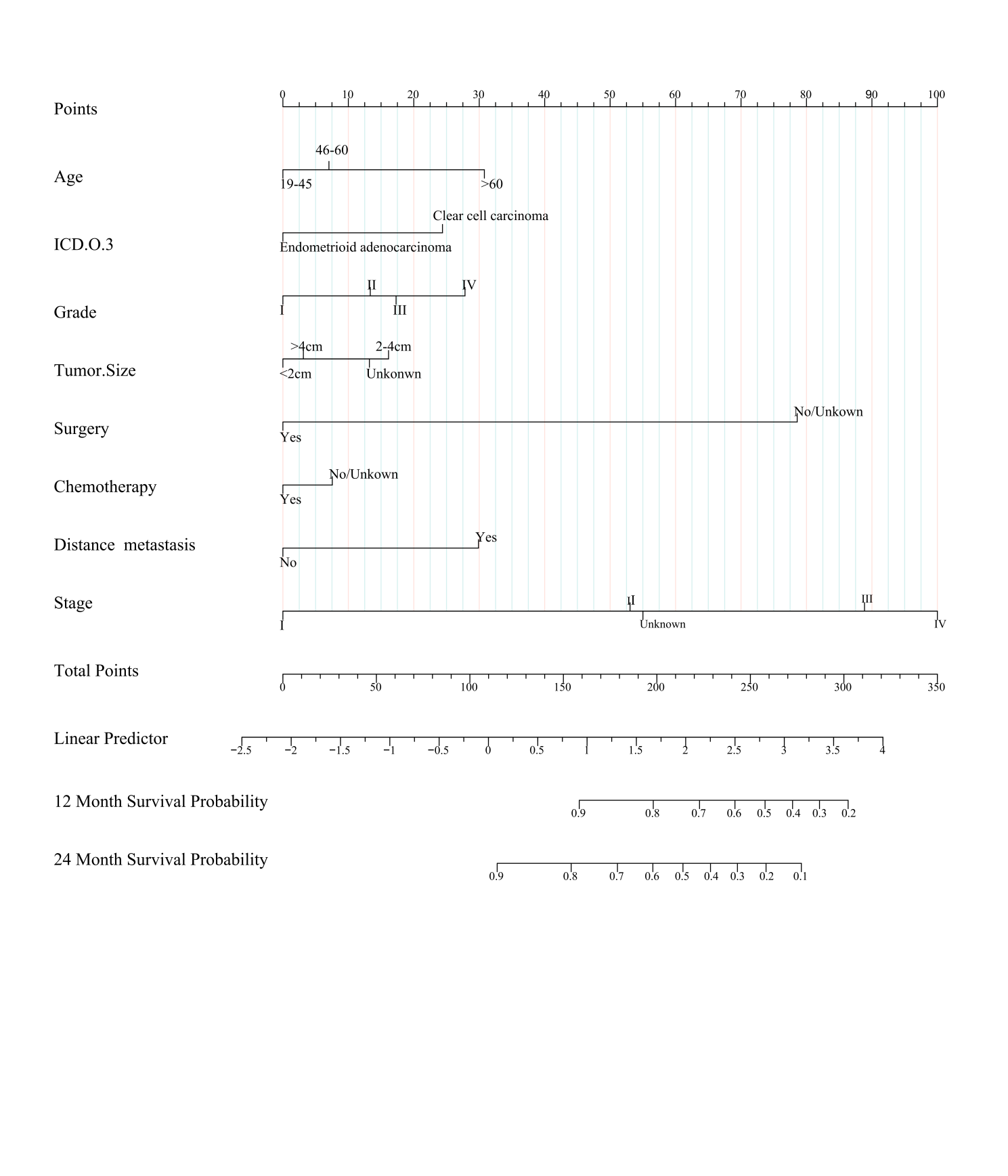
**Supplementary Figure 2.** Nomogram for predicting cancer specific survival (CSS) in patients with CCAC and CEAC using variables from a model development cohort.

Abbreviations: CSS, cancer specific survival; CCAC: cervical clear cell adenocarcinoma; CEAC, cervical endometrioid adenocarcinoma.


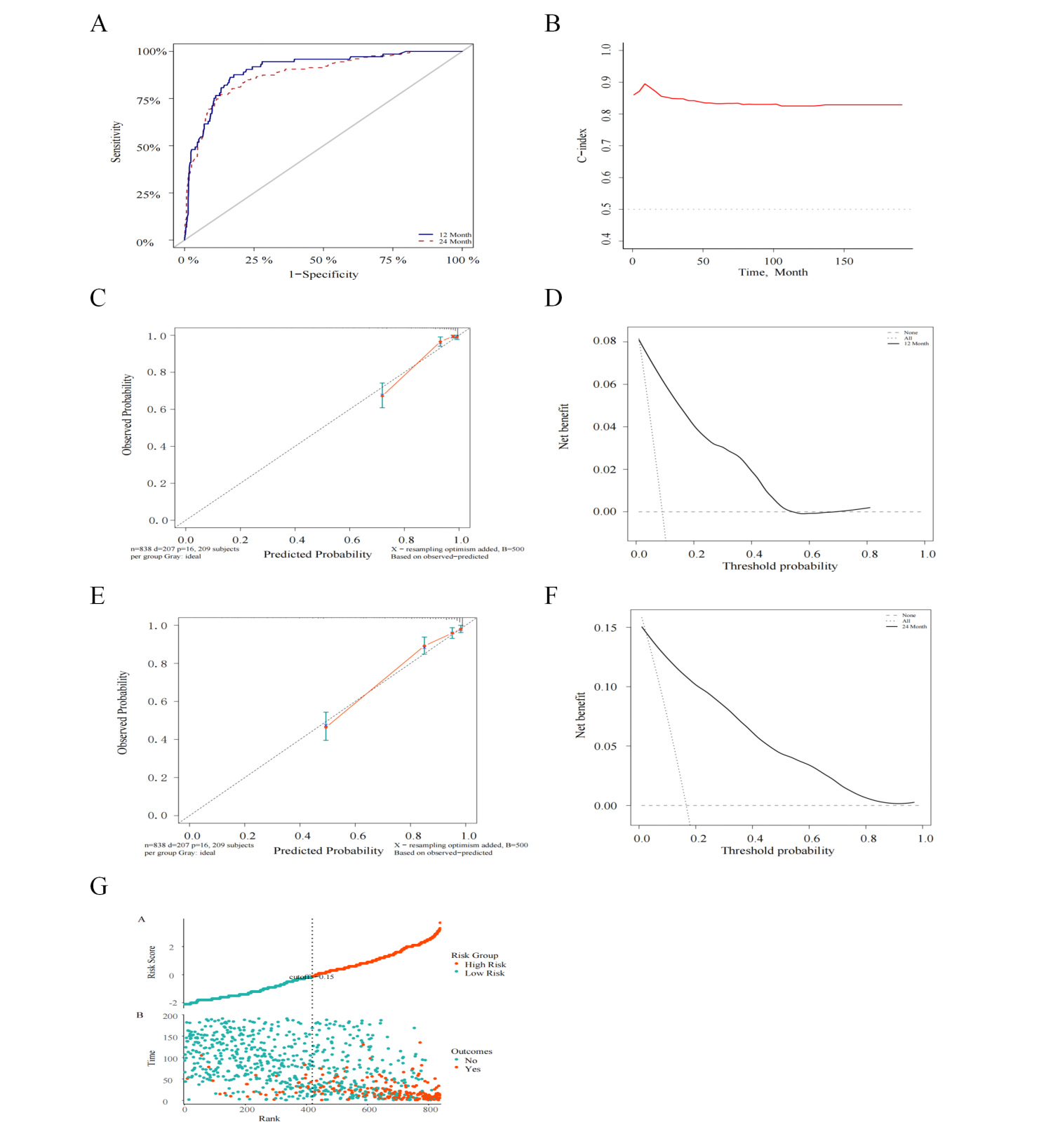


**Supplementary Figure 3.** ROC curves, calibration curves, DCA, CIC, and Risk Stratification Plot for CSS at 12 and 24 months in the model-development cohort.

The ROC curve shows that the CSS discrimination accuracy for the 12-month prediction model is 0.904 (95% CI: 0.867-0.940), while for the 24-month prediction model, it is 0.881 (95% CI: 0.847-0.914). (B) Calibration curves for CSS at different time points. (C) The close alignment of the DCA curves with the reference lines at 12 months. (D) The CIC plot for 12 months emphasizes the model’s substantial net benefit across a wide range of threshold probabilities. (E) The close alignment of the DCA curves with the reference lines at 24 months. (F) The CIC plot for 24 months highlights the model’s significant net benefit across various threshold probabilities, demonstrating its robust clinical utility. (G) Risk Stratification Plot: based on their risk scores, patients were divided into low-risk and high-risk group.

Abbreviations: ROC, Receiver Operating Characteristic; DCA, Decision Curve Analysis; CIC, Clinical Impact Curve; CSS, cancer specific survival


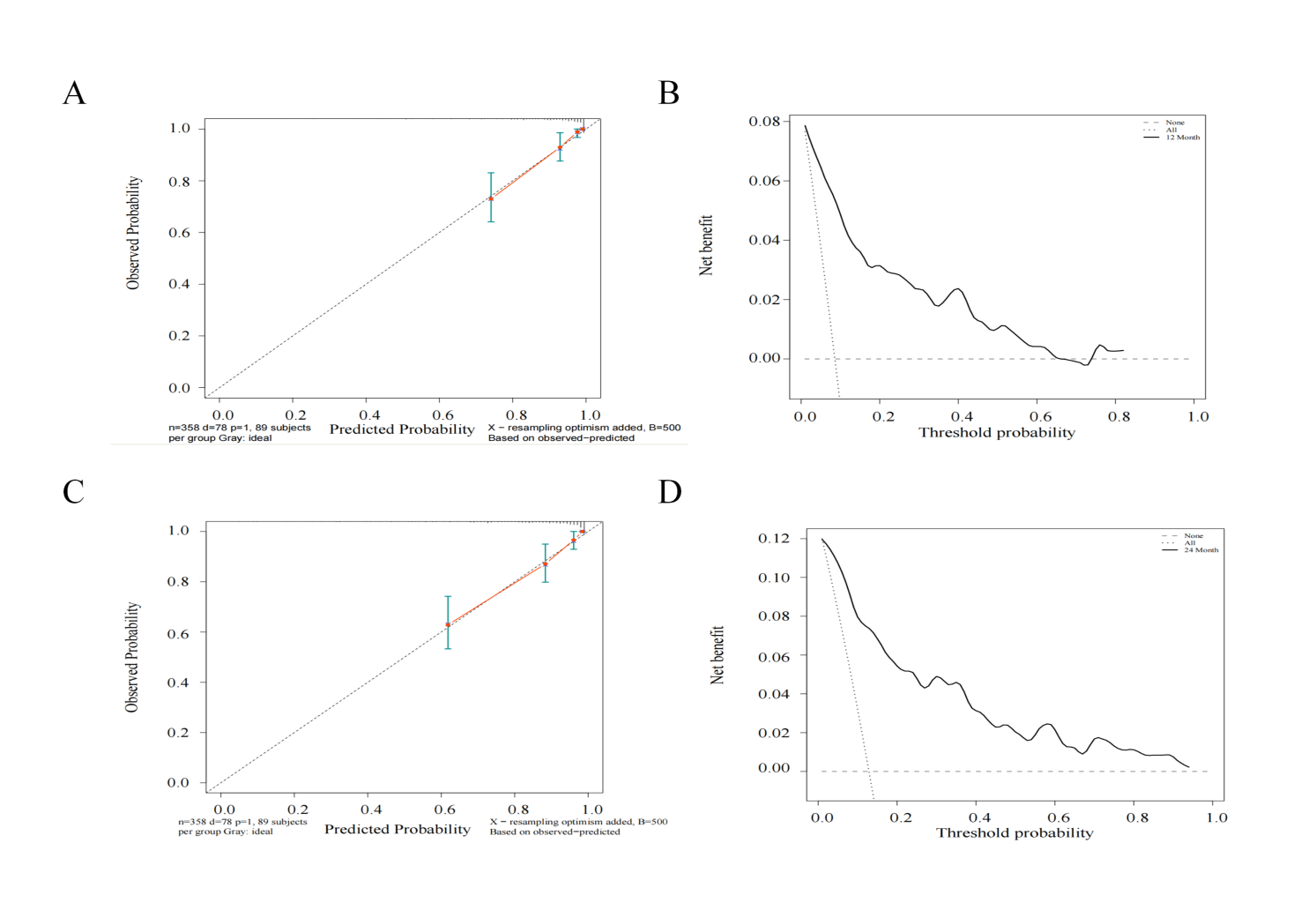


**Supplementary Figure 4.** DCA and CIC for CSS at 12 and 24 months in the internal validation cohort.

The DCA curves closely align with the reference lines at 12 months, indicating strong decision support at this time point. (B) The CIC plot for 12 months demonstrates the model’s substantial net benefit across a broad range of threshold probabilities. (C) At 24 months, the DCA curves also align closely with the reference lines, underscoring the model’s reliable decision support. (D) The CIC plot for 24 months highlights the model’s significant net benefit across various threshold probabilities, showcasing its robust clinical utility.

Abbreviations: ROC, Receiver Operating Characteristic; DCA, Decision Curve Analysis; CIC, Clinical Impact Curve; CSS, cancer specific survival


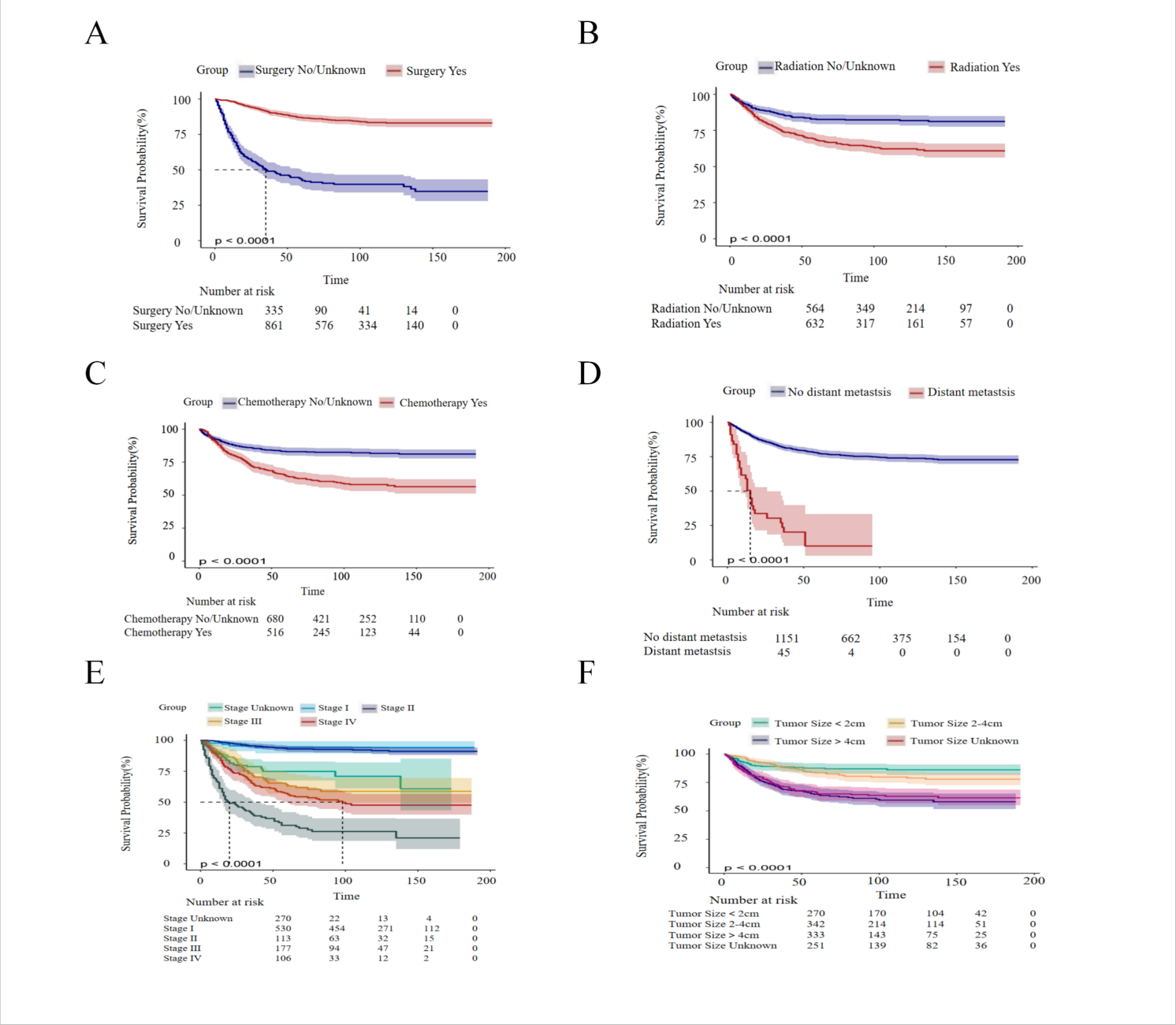


**Supplementary Figure 5.** Survival Analysis and Prognostic Factors in CCAC and CEAC

(A-C)Patients who underwent surgery had a higher overall survival rate, whereas those who received chemotherapy and radiotherapy showed a relative decrease in survival rate. This underscores the effectiveness of surgical intervention in improving prognosis for patients at an early stage.(D)Patients without distant organ metastasis have a median survival time of 190 months, whereas those with distant metastasis have a median survival of only 13 months.(E-F)Analysis indicates that early diagnosis stage and smaller tumor are strongly associated with prolonged survival.

## Supplementary Tables

**Supplementary Table 1. Univariate and multivariate analysis in the cohort.**

| Characteristics | crude.OR (95%CI) | crude.P value | adj.OR (95%CI) | adj.P value |
| --- | --- | --- | --- | --- |
| Histological type |  |  |  |  |
| Endometrioid carcinoma | ref |  |  |  |
| Clear cell adenocarcinoma | 2.52 (2.06~3.08) | <0.001 | 1.72 (1.32~2.23) | <0.001 |
| Race |  |  |  |  |
| White | ref |  |  |  |
| Others | 1.28 (1.01~1.64) | 0.044 | 1.16 (0.9~1.49) | 0.243 |
| Age |  |  |  |  |
| 19-45 | ref |  |  |  |
| 46-60 | 2.07 (1.54~2.78) | <0.001 | 1.73 (1.28~2.34) | <0.001 |
| >60 | 3.92 (2.95~5.22) | <0.001 | 2.86 (2.13~3.83) | <0.001 |
| Stage |  |  |  |  |
| Unknown | ref |  |  |  |
| I | 0.27 (0.19~0.37) | <0.001 | 0.45 (0.31~0.64) | <0.001 |
| II | 1.11 (0.77~1.59) | 0.573 | 1.01 (0.69~1.49) | 0.941 |
| III | 1.36 (0.99~1.87) | 0.059 | 1.9 (1.31~2.74) | 0.001 |
| IV | 2.74 (1.97~3.81) | <0.001 | 2.59 (1.76~3.8) | <0.001 |
| Grade |  |  |  |  |
| I | ref |  |  |  |
| II | 1.53 (1.09~2.14) | 0.014 | 1.34 (0.95~1.89) | 0.097 |
| III | 2.94 (2.1~4.11) | <0.001 | 1.43 (0.97~2.1) | 0.069 |
| IV | 3.14 (2.29~4.32) | <0.001 | 1.35 (0.93~1.96) | 0.113 |
| Tumor size |  |  |  |  |
| <2cm | ref |  |  |  |
| 2-4cm | 1.13 (0.8~1.59) | 0.477 | 1.1 (0.76~1.57) | 0.619 |
| >4cm | 2.43 (1.78~3.33) | <0.001 | 1.02 (0.72~1.45) | 0.9 |
| Unknown | 2.45 (1.78~3.38) | <0.001 | 1.38 (0.98~1.94) | 0.068 |
| Regional lymph nodes |  |  |  |  |
| Negative/Unknown | ref |  |  |  |
| Positive | 1.5 (1.15~1.96) | 0.003 | 1.08 (0.76~1.52) | 0.672 |
| Surgery |  |  |  |  |
| No/Unknown | ref |  |  |  |
| Yes | 0.19 (0.15~0.23) | <0.001 | 0.26 (0.2~0.33) | <0.001 |
| Radiation |  |  |  |  |
| No/Unknown | ref |  |  |  |
| Yes | 2.1 (1.7~2.59) | <0.001 | 1.02 (0.78~1.33) | 0.89 |
| Chemotherapy |  |  |  |  |
| No/Unknown | ref |  |  |  |
| Yes | 1.96 (1.6~2.4) | <0.001 | 0.61 (0.47~0.8) | <0.001 |
| Distance metastasis |  |  |  |  |
| No | ref | 1 | ref | 1 |
| Yes | 6.21 (4.32~8.94) | <0.001 | 1.76 (1.15~2.69) | 0.01 |
